# Prevalence, components and associated demographic and lifestyle factors of the metabolic syndrome in type 2 diabetes mellitus

**DOI:** 10.1186/2251-6581-13-80

**Published:** 2014-07-15

**Authors:** Victor Mogre, Zenabankara S Salifu, Robert Abedandi

**Affiliations:** 1 Department of Human Biology, School of Medicine and Health Sciences, University for Development Studies, P. O. Box TL 1883, Tamale, Ghana; 2 Department of Allied Health Sciences, School of Medicine and Health Sciences, University for Development Studies, P. O. Box TL 1883, Tamale, Ghana

**Keywords:** Metabolic syndrome, Type 2 diabetes mellitus, Clinical factors, Demographic factors, Tamale, Ghana

## Abstract

**Background:**

Adults with the metabolic syndrome (MetS) are twice as likely to die from and three times as likely to have a heart attack or stroke compared with people without the syndrome. About 70-80% of type 2 diabetes mellitus (type 2 DM) patients are diagnosed with the MetS. Investigating the occurrence of the MetS in type 2 DM patients is critical for cardiovascular disease prevention. We evaluated the prevalence and components of the MetS and its associated clinical and demographic factors in a Ghanaian adult population with DM 2.

**Methods:**

This cross-sectional study was conducted among 200 previously diagnosed type 2 DM patients receiving care from an outpatient clinic of the Tamale Teaching Hospital, Ghana. Anthropometric measurements of waist circumference (cm), weight (Kg) and height (m) were measured appropriately. Clinical data were obtained from the personal health record files of the participants. MetS was defined according to the International Diabetes Federation criteria.

**Results:**

The prevalence of MetS was 24.0% (n=48). The prevalence was higher in women (27.3%, n= 42) compared to men (13.0%, n=6). The commonest occurring components of the MetS included abdominal obesity (77.0%) and elevated FPG (77.0%) denoting uncontrolled diabetes. The prevalence of elevated BP was found to be 44.0%(n=88) and was higher in men (56.5%) than in women (40.3%). Factors that were found to be associated to the MetS were being overweight/obese (Crude OR = 2.9, 95% CI = 1.43 – 5.90, p=0.004), ever tried to lose weight (Crude OR = 2.5, 95% CI = 1.24 – 4.94, p=0.015) and having diabetes for over 5 years (Crude OR = 11.3, 95% CI = 5.26 – 24.08, p<0.001). Other factors that were associated to the MetS were current smokers (Crude OR = 6.8, 95% CI = 1.21- 38.49, p=0.030) and alcohol drinkers (Crude OR = 3.1, 95% CI = 1.23 – 7.65, p=0.018).

**Conclusion:**

A comparatively low prevalence of the MetS was found. More females than males had the MetS. Uncontrolled diabetes and abdominal obesity were prevalent. The factors identified by our univariate logistic regression model were not significant predictors of the MetS in our multivariate model.

## Background

The metabolic syndrome (MetS) as defined by the International Diabetes Federation (IDF) is a constellation of the most dangerous risk factors of heart attack including diabetes and raised fasting plasma glucose, abdominal obesity, high cholesterol and high blood pressure [1-3]. Globally, 20-25% of the adult population has been estimated to have the MetS and are twice as likely to die from and three times as likely to have a heart attack or stroke compared with people without the syndrome [4].

The underlying cause of the MetS has been linked to various forms of glucose metabolism including insulin resistance [5], glucose intolerance [6] and abnormalities in glucose metabolism [7]. Almost 70-80% of the population with diabetes mellitus is diagnosed with the MetS [8,9]. Recently a study in Nigeria reported the prevalence of the MetS in type 2 diabetes mellitus (type 2 DM) patients to be 86% [10]. Another study in Cameroon found a prevalence of 71.7% based on the IDF criteria and 60.4% on the NCEP-ATP III in persons with type 2 DM [11]. Evidently, individuals with the MetS have a fivefold greater risk of developing type 2 DM [4].

The co-occurrence of type 2 DM and the MetS potentiates the cardiovascular risk associated with each of the two conditions [11]. Evaluating the MetS in persons with diabetes is thus critical for the purposes of cardiovascular disease prevention. It is even more so in this current situation of the rising prevalence of type 2 DM in Sub-Saharan Africa. Studies on the prevalence of the MetS among type 2 DM patients are limited in a developing country like Ghana and in other Sub-Saharan countries.

Accordingly, the aim of this study was to assess the prevalence and components of the MetS among type 2 DM patients in Tamale, Ghana. Furthermore, we investigated the demographic and lifestyle factors associated to the MetS in this sample population.

## Materials and methods

### Participants

Two hundred previously diagnosed type 2 DM patients receiving care from the outpatient diabetes clinic of the Tamale Teaching Hospital, Ghana were recruited to participate in this cross-sectional study. As described elsewhere [12], all previously diagnosed diabetes patients based on the WHO criteria [13], that sought for care from the Hospital’s diabetes clinic, during the study period were eligible to participate in the study. Two hundred and fifteen participants were approached; 200 of them consented to the study, yielding a response rate of 97.6%.The Tamale Teaching Hospital is the major referral centre for a catchment population of 4, 228,116 inhabitants. Tamale is the capital city of the Northern region of Ghana and located about 600km north of Accra, the capital city of Ghana. It lies between latitude 9°22′N and longitude 0°50′W.

Informed consent was obtained from each participant. The ethics committees of the School of Medicine and Health Sciences of the University for Development Studies and the Tamale Teaching hospital approved the study.

### Inclusion criteria

Participants aged ≥ 30 years; clinical determined type 2 DM and duration of diabetes of at least 1 year were qualified to participate in the study.

### Exclusion criteria

Participants aged < 30 years, Pregnant and lactating mothers were excluded from the study. Also participants with a history of heart failure, type 1 DM, myocardial infarction, acromegaly, hypothyroidism, hypogonadism and any other chronic diseases; patients on prolong steroid use, and those who were on active drug treatment for obesity at the time of admission were excluded from the study.

### Anthropometric measurements

Anthropometric measurements of waist circumference (cm), weight (Kg) and height (m) were measured. Waist circumference (WC) was measured midway between the inferior angle of the ribs and the suprailiac crest [14]. It was measured to the nearest 1 cm using a non-stretchable fibre-glass measuring tape (Butterfly, China). During the measurement, participants stood in an upright position, with arms relaxed at the side, feet evenly spread apart and body weight evenly distributed in accordance with the WHO expert consultation report on waist circumference and waist-hip ratio [14]. Weight was measured to the nearest 0.1 kg using a UNICEF electronic scale manufactured by seca. Height was measured using a wall-mounted microtoise and recorded to the nearest 0.5 cm. BMI was calculated as weight (kg)/height^2^ (m^2^) and used to categorize BMI-measured weight status: underweight (BMI ≤ 18.5), normal (BMI 18.5–24.9), overweight (BMI 25.0–29.9) and obese (BMI ≥30) [15].

### Demographic and clinical parameters

Participants’ clinical parameters of systolic and diastolic blood pressures and Fasting Plasma Glucose (FPG) were recorded from their personal health record files. Parameters such as gender, age and duration of diabetes were also obtained from the participants by means of a pre-designed questionnaire. Impaired Fasting Glycaemia (IFG) was defined as FPG ≥ 6.1 mmol l^-1^[13]. Hyperglycaemia was determined by a FPG ≥ 7.0 [13]. IFG and hyperglycaemia were combined to denote uncontrolled diabetes. Smoking habits, coffee and alcohol drinking were also collected using the questionnaire.

### Definition of the MetS

The Metabolic syndrome (MetS) was defined according to the International Diabetes Federation (IDF) consensus worldwide definition of the MetS [3]. According to the new IDF definition; for a person to be defined as having the MetS they must have: Central obesity (defined as waist circumference (Men ≥ 94 cm and Women ≥ 80 cm) plus any two of the following four factors: Raised triglycerides ≥ 150 mg/dL (1.7 mmol/L) or specific treatment for this lipid abnormality; Reduced HDL cholesterol < 40 mg/dL (1.03 mmol/L) in males, < 50 mg/dL (1.29 mmol/L) in females or specific treatment for this lipid abnormality; Raised blood pressure (BP): systolic BP ≥ 130 or diastolic BP ≥ 85 mm Hg or treatment of previously diagnosed hypertension and; Raised fasting plasma glucose (FPG) ≥ 100 mg/dL (5.6 mmol/L), or previously diagnosed type 2 diabetes. If above 5.6 mmol/L or 100 mg/dL, OGTT is strongly recommended but is not necessary to define presence of the syndrome. We used central obesity, raised blood pressure and raised FPG to define the MetS in this study.

### Statistical analysis

Data were entered into Microsoft Excel 2007 and analyzed using two statistical software packages: Graphpad Prism version 5.00 (GraphPad software, San DiegoCalifornia USA, http://www.graphpad.com) for windows and the statistical package for the social sciences (SPSS) (version 18) for windows. All continuous data were expressed as mean ± S.D and compared using student t-test. All categorical data were expressed as frequencies and proportions and compared using Fisher’s exact test. Statistical significance was assumed at a p-value of < 0.05. Univariate and multinomial logistic regression analysis were conducted to identify associated factors to the MetS.

## Results

The background characteristics of the participants stratified by gender are presented in Table 1. These 200 participants with type 2 DM had a mean ± SD age of 56.2 ± 12.13 years in which women were significantly (p = 0.031) older than men. The mean duration of diabetes was 5.23 ± 5.00 and significantly higher in men than in women. The prevalence of current smokers and alcohol drinkers were found to be 3.0% (n =6) and 11.0% (n = 22) respectively. Half (n =100) of the participants took coffee.

**Table 1:** Background characteristics of the participants stratified by gender

**Variable**	**Total (n = 200)**	**Men (n = 46)**	**Women (n = 154)**	**P value**
Age (years)	56.2 ± 12.13	52.83 ± 10.89	57.22 ± 12.33	0.031
Duration of diabetes (years)	5.23 ± 5.00	6.37 ± 4.96	4.88 ± 4.98	0.023
**Educational status**				
Low (%)	132 (66.0%)	26 (56.5%)	106 (68.8%)	0.156
**Married**				
Yes (%)	48 (24.0%)	12 (26.1%)	36 (23.4%)	0.698
**Alcohol consumption**				
Yes (%)	22 (11.0%)	0 (0.0%)	22 (14.3%)	0.003
**Smoking**				
Yes (%)	6 (3.0%)	0 (0.0%)	6 (3.9%)	0.340
**Coffee consumption**				
Yes (%)	100 (50.0%)	18 (39.1%)	82 (53.2%)	0.130

The prevalence and components of the MetS stratified by gender are presented in Table 2. The mean ± SD body mass index (BMI) of all the participants was 23.86 ± 4.64 Kg/m^2^ with no significant differences between men and women. Significantly (p < 0.001), women had larger mean WC than men with an abdominal obesity prevalence of 26.1% (n = 12) in men and 92.2% (n = 142) in women. However, men had higher mean levels of systolic and diastolic blood pressures than women. Generally, the mean ± SD fasting plasma glucose (FPG) was 7.94 ± 2.81 and higher in men than in women even though the differences were not significant. The prevalence of uncontrolled diabetes was 77.0% (n = 154) from which 29.0% (n = 46) had impaired fasting glycaemia (IFG) and the rest had hyperglycaemia (71.0%, n = 108). The prevalence of metabolic syndrome was found to be 13.0% (n = 6) in men and 27.3% (n = 42) in women. However, the differences were not significant (p = 0.051) when the prevalence of metabolic syndrome was stratified by gender using Fisher’s exact test.

**Table 2:** Prevalence and components of the metabolic syndrome stratified by gender

**Variable**	**Total (n = 200)**	**Men (n = 46)**	**Women (n = 154)**	**P value**
Metabolic Syndrome (%)	48 (24.0%)	6 (13.0%)	42 (27.3%)	0.051
WC (cm)	95.99 ± 15.63	85.17 ± 13.98	99.22 ± 14.64	< 0.001
Abdominal obesity (%)	154 (77.0%)	12 (26.1%)	142 (92.2%)	< 0.001
Systolic BP (mmHg)	122.80 ± 16.17	127 ± 12.09	121.6 ± 17.04	0.014
Diastolic BP (mmHg)	84.50 ± 13.85	88.26 ± 11.80	83.38 ± 14.24	0.023
Hypertension (%)	88 (44.0%)	26 (56.5%)	62 (40.3%)	0.063
FPG (m/mol)	7.94 ± 2.81	8.07 ± 2.77	7.90 ± 2.83	0.904
Uncontrolled diabetes (%)	154 (77.0%)	34 (73.9%)	120 (77.9%)	0.556
IFG (%)	46/154 (29.9%)	12/34 (35.3%)	34/120 (28.3%)	0.525
Hypergglycaemia (%)	108/154 (70.1%)	22/34 (64.7%)	86/120 (71.7%)	
BMI (Kg/m^2^)	23.86 ± 4.64	24.28 ± 4.56	23.74 ± 4.66	0.716
Overweight/obese (%)	64 (32.0%)	16 (34.8%)	48 (31.2%)	0.719

BMI = Body mass index, WC = Waist circumference, BP = Blood pressure, FPG = Fasting plasma glucose, IFG = Impaired Fasting Glycaemia.

Shown in Table 3 are the results of a univariate analysis of factors affecting the MetS. Factors that were found to be associated to the MetS were being overweight/obese (Crude OR = 2.9, 95% CI = 1.43 – 5.90, p = 0.004), ever tried to lose weight (Crude OR = 2.5, 95% CI = 1.24 – 4.94, p = 0.015) and having diabetes for over 5 years (Crude OR = 11.3, 95% CI = 5.26 – 24.08, p < 0.001). Furthermore, participants who were current smokers had a 6.8 risk of the MetS. In addition, participants who were current drinkers (Crude OR = 3.1, 95% CI = 1.23 – 7.65, p = 0.018) of alcohol were more likely to develop the MetS compared to those who did not drink alcohol.

**Table 3:** Univariate analysis of lifestyle demographic factors affecting the MetS

**Variable**	**n/N**	**Rate of metabolic syndrome**	**OR (95% CI)**	**P value**
**Gender**				
Men	6/46	13.0%	1	1
Women	42/154	27.3%	0.4 (0.16 - 1.01)	0.051
**Age (years)**				
≤ 40	6/20	30.0%	1	1
> 40	42/178	23.6%	1.4 (0.50 - 3.84)	0.583
**Overweight/obese**				
Yes	20/48	41.7%	1	1
No	28/142	19.7%	2.9 (1.43 - 5.90)	0.004
**Married**				
Yes	36/152	23.7%	1	1
No	12/48	25.0%	0.9 (0.44 - 1.98)	0.848
**Educational status**				
Low	36/132	27.3%	1	1
High	12/68	17.6%	1.8 (0.84 - 3.64)	0.163
**Ever tried to lose weight**				
Yes	20/54	37.0%	1	1
No	28/146	19.2%	2.5 (1.24 - 4.94)	0.015
**Duration of diabetes**				
≤ 5 years	12/132	9.1%	11.3 (5.26 - 24.08)	< 0.001
> 5 years	36/68	52.9%	1	1
**Smoke**				
Yes	4/6	66.7%	1	1
No	44/194	22.7%	6.8 (1.21 - 38.49)	0.030
**Alcohol consumption**				
Yes	10/22	45.5%		
No	38/178	21.3%	3.1 (1.23 - 7.65)	0.018
**Coffee consumption**				
Yes	22/100	22.0%		
No	26/100	26.0%	0.8 (0.42 - 1.54)	0.620

As shown in Table 4, none of the factors identified by our univariate logistic regression model were significant in the MetS from our multinomial logistic regression model.

**Table 4:** Multinomial logistic regression of clinical and demographic factors affecting the MetS

**Independent variables**	**B**	**OR (95% CI)**	**P value**
Intercept	1.47		<0.001
**Overweight/obese**			
Yes	0.66	1.9 (0.96 – 3.94)	0.067
No	1	1	1
**Ever tried to lose weight**			
Yes	-0.80	0.4 (0.20 – 1.02)	0.055
No	1	1	1
**Duration of diabetes**			
≤ 5 years	-0.63	0.5 (0.24 – 1.21)	0.134
>5 years	1	1	1
**Smoking**			
Yes	-1.07	0.34 (0.05 – 2.55)	0.295
No	1	1	1
**Alcohol consumption**			
Drinks alcohol	-0.12	0.9 (0.26 – 3.05)	0.850
Does not drink alcohol	1	1	1

Cox and Snell = 0.070, Nagelkerke = 0.104, McFadden = 0.066.

## Discussion

We report here a comparatively low prevalence of the metabolic syndrome (MetS) in an adult Ghanaian population with diabetes mellitus type 2 (DM).

Studies on the prevalence of the MetS among diabetes patients in Sub-Saharan Africa are limited and the few ones that are available are variable with rates similar to the ones reported in developed countries [16-18]. The MetS prevalence of 24.0% reported among type 2 DM patients in our study can be said to be among the lowest in literature and is only comparable to the 25.2% prevalence of MetS among type 2 DM patients in southern Nigeria using the WHO criteria [19]. A study among diabetes patients in Cameroon found the prevalence of the MetS defined by IDF to be 71.7% and 60.4% defined by NCEP-ATP III [11]. In Nigeria, Adediran et al., [20] reported an overall MetS prevalence of 51%, 44% in men and 56% in women in a group of 408 type 2 DM individuals at a University Teaching Hospital in Lagos, Nigeria based on the WHO criteria. In another University teaching Hospital in Northern Nigeria, Isezuo and Ezunu reported a MetS prevalence ranging from 54–59% among diabetic patients in 2002 [21,22]. In Zimbabwe, Makuyana et al., [23] reported a 43% prevalence rate of MetS in 109 diabetic subjects in a tertiary care diabetes clinic [24]. In some other parts of Africa, rates of 66.8%, 85.5%, 74% among men and 87.1%, 79.7%, 93% among women, according to the NCEP-ATP III, WHO and IDF criteria, respectively, reported in Seychelles [25] and rates of 46% and 74% in Black and White South Africans respectively based on the IDF criteria [24]. The prevalence of the syndrome of our study is also lower than rates reported among Caucasians [26-28] with type-2 diabetes mellitus. From the above, it can be said that the prevalence rates found in our study are among the lowest so far reported in sub- Saharan Africa. The differences could be due to the variable criteria used in defining the MetS as well as ethnic differences. In addition it could be due to the fact that we did not include HbA1c, HDL and triglycerides as criteria for defining the MetS.

Another important finding of our study was that the prevalence of the MetS was found to be higher in women than in men. This is in agreement with several studies conducted among type 2 DM patients in Sub-Saharan Africa [10,11,21] and other developing countries [29-32]. However, several other studies especially in developed countries have reported contrary findings [33-36].The high prevalence of the MetS found among women in our study could be due to the fact that a significant proportion of women had abdominal obesity which is one of the components of the MetS of the IDF criteria used in this study. Similar reasons have also been given by several studies that have reported higher prevalence of the MetS in women than in men [29,37]. Women should become a focus of high risk target screening, and attempts should be made to normalize each component of the MetS so as to minimize the risk of cardiovascular diseases [29].

Variable rates of the components of the MetS were found. Similar to the findings of Ogbera [10] and Kelliny et al [25] abdominal obesity was one of the components of the MetS that occurred frequently. However, contrary to their findings it was more prevalent in women than in men. They found comparable rates between men and women. The high prevalence of central obesity found in our study is not surprising. Abdominal obesity is the principal factor for defining the MetS and precedes the other components of the MetS. Obesity is associated with insulin resistance and contributes to hypertension, high serum cholesterol, low HDL-c and hyperglycaemia, and is independently associated with higher CVD risk [38-40].

Another component of the MetS that was also found to be prevalent in our study was hyperglycaemia and impaired fasting glycaemia (IFG) denoting uncontrolled diabetes. Same as the prevalence rate of the abdominal obesity, 71.0% of the participants had hyperglycaemia, a FPG ≥ 7 mmol^-1^ and the rest had IFG a FPG ≥ 6.1. This is a worrying finding in the sense that FPG values above 6.1 mmol^-1^ are associated with a progressively greater risk of developing micro– and macro-vascular complications [41-43].

From our univariate analysis, participants who were overweight/obese measured by BMI had a 2.9 risk of developing the MetS. Consistent with our findings Ogbera [10], found that participants who had the MetS had higher body mass indices. Also, Kip et al., [44] found that the metabolic status was strongly related to BMI, with 28% of women with normal BMI being dysmetabolic compared with 55% among overweight women and 76% among obese women in a study among women in the US. In as much as the association between general overweight/obese was not significant in predicting the MetS in our multinomial logistic regression model, its management should be considered in the treatment and management of DM 2 patients.

Another important finding of our study was that participants who had diabetes for over 5 years were found to have an 11.3 risk of developing the MetS from our univariate model. This is contrary to the findings of Shimajiri et al., [36] in which the prevalence of the MetS decreased along with an increase in the duration of diabetes. Another study by Abdul-Ghani et al., [30] also found an association of the MetS with less duration of diabetes. The reasons provided by the authors of the two studies for the decrease in the MetS with an increase in the duration of diabetes were: decreased BMI as a result of medical intervention and better metabolic control in diabetic patients due to increasing awareness with the longer duration of diabetes. Even though, the association of the MetS with higher duration of diabetes was found to be non-significant in our multinomial logistic regression model, the association could be as a result of lack of awareness as well as inadequate access to quality medical care or lack of quality care.

Participants with the MetS were more likely to have ever tried to lose weight, current smokers and drinkers of alcohol. However, these factors were not significant predictors of the MetS in our multinomial logistic regression model. Further extensive studies should be conducted into identifying the effects of these factors on the MetS among DM 2 patients.

Our study is not without limitations. This was a cross-sectional study and cannot be used to establish causality. It however provides a basis upon which future prospective studies could be executed. FPG and blood pressure values were obtained secondarily from the personal health files of the diabetes patients. Although, all care was taken to record the values to minimize errors, misreporting might have occurred. The method of assessment of the effect of coffee and alcohol on adiposity is limited in the sense that we did not consider the frequency and quantity of coffee/alcohol consumed. However, the findings of this study provide first-hand information on the effect of coffee and alcohol on the MetS and form a basis for further studies. Moreover, we did not measure HbA1c, HDL and triglycerides in this study due to the unavailability of funds. This may affect the frequency of MetS. Notwithstanding these limitations, this preliminary study was able to reveal important aspects of this clinical syndrome among diabetic patients in Ghana.

## Conclusion

A comparatively low prevalence of the MetS has been found in this study. A higher number of women than men had the MetS. A high prevalence of abdominal obesity was found. Majority of the participants had uncontrolled diabetes. Factors including being: generally overweight/obese, ever tried to lose weight, having longer duration of diabetes, current smokers and alcohol drinkers, that were found to be associated with the MetS in our univariate analysis were not significant predictors in our multinomial logistic regression model.

## Abbreviations

ATP: National Cholesterol Education Program-Adult Treatment Panel III; WHO: World Health Organization; IDF: International diabetes federation.

## Competing interests

The right of we the authors to examine, analyze, and publish the data of the research is not infringed upon by any contractual agreement or conflict of interest.

## Authors’ contributions

VM: Conducted analysis and Interpretation of the data, drafting of the manuscript and critical revision of the manuscript. ZSS: Concept and Design. RA: Data Acquisition. All authors read and approved the final manuscript.
